# The Impact of Thyme, Rosemary and Basil Extracts on the Chemical, Sensory and Microbiological Quality of Vacuumed Packed Mackerel Balls

**DOI:** 10.3390/foods11182845

**Published:** 2022-09-14

**Authors:** Esra Balikçi, Yesim Özogul, Nikheel Bhojraj Rathod, Fatih Özogul, Salam A. Ibrahim

**Affiliations:** 1 Department of Gastronomy and Culinary Arts, Faculty of Tourism, YozgatBozok University, Yozgat 66900, Turkey; 2 Department of Seafood Processing Technology, Faculty of Fisheries, University of Cukurova, Adana 01330, Turkey; 3 Department of Postharvest Management of Meat, Poultry and Fish, Post Graduate Institute of Post Harvest Management (Dr. Balasaheb Sawant Konkan Krishi Vidyapeeth), Roha 402 116, Raigad, Maharashtra State, India; 4 Food Microbiology and Biotechnology Laboratory,171 Carver Hall, College of Agriculture and Environmental Sciences, North Carolina A & T State University, Greensboro, NC 27411-1064, USA

**Keywords:** value addition, biochemical quality, microbiology, shelf-life

## Abstract

The effect of natural extracts (0.05%) and vacuum packaging on the sensory, chemical, and microbiological quality of mackerel balls were evaluated at refrigerated (4 ± 2 °C) storage. Natural extracts thyme (38.13 mg GAE/g), rosemary (81.85 mg GAE/g) and basil (21.08 mg GAE/g) were evaluated. Natural extracts imparted stability to lipids (TBA, FFA, and PV), and the ability was further improved by vacuum packaging. Biochemical changes (TVB-N, pH) and microbiological quality (total viable count) were also retained. Control samples packed under vacuum were found to cross over acceptable limits on day 28. Based on sensory quality evaluation, samples treated with rosemary and thyme extracts showed superior sensory quality over control, whilebasil-treated samples were not found acceptable at day 28. Consequently, the inclusion of thyme and rosemary extracts exhibits preservative quality when combined with vacuum packaging, retaining biochemical, microbial, and sensory quality.

## 1. Introduction

Mackerel is an important pelagic fish with global importance since this fish is rich in proteins, fatty acids, vitamins, and minerals [1]. Mackerel is processed into different value-added forms to further increase its commercial value. The development of favorable products is an important issue in fisheries, imparting convenience to the consumer with higher returns [2]. Amongst value-added products in fisheries, fish balls are regarded as an important favorable product [3]. Fishes are rich in nutrients such as proteins and lipids (unsaturated fatty acids) with the ability to support good health and mental wellbeing [4,5]. Fishes are highly perishable in nature, being degraded by the action of microorganisms and oxidation. Microorganisms, spoilage, and pathogenic by-products of lipid oxidation generate products responsible for fish spoilage and are responsible for food borne illness [6,7,8]. Considering the importance of oxygen in inducing oxidation of lipids [6,7], vacuum packaging technology is constantly being integrated with other technologies for the preservation of foods [9]. Furthermore, considering the highly perishable nature of fish and fishery products, the wide range of preservatives (antioxidants and antimicrobials) from synthetic sources are used for the preservation of aquatic food products [6]. The additions of synthetic preservatives have been known to have negative effects, while some synthetic preservatives have been banned in some countries [6]. Therefore, the preservatives from natural sources are constantly being evaluated for the preservation of aquatic food products.

Natural preservatives derived from plant sources exhibit diverse mechanisms for the inactivation of microorganisms and inhibition of lipid oxidation, with no toxic residue at a lower cost [10,11]. Plant parts are rich in phenolic constituents that are known to exhibit preservative abilities [6,10]. Due to their superb preservation capacity, they are extensively evaluated in combination with several non-thermal processing technologies such as cold plasma, pulsed electric field, and high hydrostatic pressure [11,12]. Several packaging interventions, such as vacuum packaging, have exhibited quality retention and shelf life extension in aquatic food products by limiting the exposure of oxygen and the growth of microorganisms [13]. Storage temperature for aquatic food products has a pivotal role during preservation since temperatures below 5 °C are known to reduce muscle degradation.

Considering the importance of inclusion of natural additives in foods due to their high bioactivity [10], several studies have reported the inclusion of natural extracts from thyme, rosemary and basil for the preservation of foods [14,15,16]. Amongst natural preservatives, rosemary, thyme, and basil have been widely applied and reported for their preservation abilities due to antioxidant and antimicrobial activity as well as higher total phenolic content [14,17]. Additionally, they have improved the sensory and nutritional qualities in fishery products [15,16,18,19].

Therefore, the objective of this study was to investigate the impact of the application of thyme, rosemary, and basil extracts combined with vacuum packaging to improve the storage quality of mackerel balls at chilled storage (4 ± 2 °C).

## 2. Materials and Methods

### 2.1. Plant Extract

Extracts from rosemary (*Rosmarinus officinalis* L.), thyme (*Thymbra Spicata*), and basil (*Ocimumbasilicum* L.) were obtained by the methods given by Chen and others [20]. The total phenolic contents of the extracts were assayed by the method given by Singleton and others [21]. The total phenolic contents for rosemary, thyme, and basil was found to be 81.85, 38.13, and 21.08 mg GAE/g, respectively.

### 2.2. Preparation of Samples

Atlantic mackerel (*Scomberomorus scombrus*) was procured locally (Pakyürek, Adana). Prior to cooking, the fishes were thawed in a refrigerator, followed by gutting and washing. For flesh separation (from skin and bones), the fishes were cooked in boiling water for 5 min and separated manually. Afterwards, the flesh was divided into four parts for treatments (control, rosemary esxtract−0.05%, thyme extract−0.05%, and basil extract−0.05%) for further making of the fish balls, as prescribed by López-Caballero and others [22], with minor modification. The fish balls were made up of 0.5% salt, 5% water, 2% egg white (Lick, Turkey), and 10% wheat starch (Kent, Istambul, Turkey) were added to minced fish, mixed thoroughly using a mixer (Easy Max Compact, France), and then shaped into a fish ball (3 cm, 15 ± 1.2 g) by hand. The prepared meatballs contained 53.72% moisture, 19.23% protein, 12.53% lipid, and 3.32% ash, suggesting a high nutritional quality/value. Prepared fish balls were packed in 90 µm thick polyamide-based packages (Polinas, Manisa, Turkey) with a vacuum packaging machine (Reepack RV50, Bergamo, Italy). Every package contained 10 fish balls and was stored at 4 ± 2 °C for 28 days. Sensory, chemical, and microbiology analyses were carried out to determine the quality of the fish ball. Data were obtained from three packages of fish balls.

### 2.3. Sensory Evaluation

For analysis, the fish balls were subjected to sensory evaluation (appearance, color, odor, and general acceptability) in the raw [20] and cooked state [23]. The evaluations were conducted by a team of six trained panellists, under daylight conditions at 24 °C. For the cooked sample analysis, samples were fried in sunflower oil (185 °C) for 2–3 min. The sensory evaluation was carried out using a 5-point scale for raw fish balls, where a score of 5 represented “like very much”, 4 suggested “neither like or dislike”, and a score of 2–1 was regarded as “dislike”. A 9 point scale was used for the evaluation of the cooked fish ball samples where 9 indicated “very good quality”, 7–8 indicated“good quality”, 5–6 as “acceptable limit”, and 1–4 suggested“bad or unacceptable”.

### 2.4. Chemical Quality Analysis

The Kjeldahl method was employed for the estimation of protein content using a digestion system from BÜCHI Labortechnic, Flawil, Switzerland and Kjeltecc distillation unit (B-324) from BÜCHI Labortechnic, Flawil, Switzerland (AOAC, 1998). For the conversion factor (from nitrogen to protein), 6.25 was used. Lipid content was estimated using the method given by Bligh and Dyer [24]. The ash level was found as described by the AOAC Method 920.153 (2002), briefly by ashing a burner-charred sample at 550–600 °C using a muffle furnace, with the moisture level being determined by the AOAC Official method 950.46 (2002) at 100–102 °C for 16–18 h using an air oven. The peroxide value (PV) indicator of lipid quality was measured by the AOCS Method Ja 8-87 (1994) and expressed as meq of oxygen per kg of lipid. The total volatile base nitrogen (TVB-N) content of samples was carried out by the method given by Antonocopoulus [25], and the results were expressed as mg of TVB-N per kilogram of muscle, suggesting the level of decomposition. Free fatty acid analysis (FFA) was obtained by the AOCS Official Method (Ca 5a-40, 1997) and expressed as a percentage of oleic acid, suggesting the rate of hydrolysis in lipids. The thiobarbituric acid (TBA) content was determined by the method of [26] and expressed as TBA value in milligrams of malondialdehyde (MA) per kilogram of flesh.

### 2.5. Microbiological Analyses

The sample (10 g) was homogenized for 2 min in the stomacher by adding 90 mL of Ringer’s solution. The samples were serially diluted (10^−8^) followed by pipetting 0.1 mL from each dilution onto Petriplates containing plate count agar and incubated (30 °C) for 2 days. Following incubation, the colonies with characteristics were counted and enumerated to arrive at a log cfu/g [2].

### 2.6. Statistical Analysis

Data was subjected to statistical analysis using the SPSS software V22 (SPSS, Chicago, IL, USA). The data of control, thyme, rosemary and basil groups were analysed by analysis of variance (ANOVA) and Duncan’s multiple range tests.

## 3. Results and Discussion

### 3.1. Sensory Evaluation

Sensorial quality changes in raw and cooked fish balls during the storage period are listed in Table 1 and Table 2, respectively. Reduced attributes (appearance, color, odor, and general acceptance) were observed for 28 days for raw fish ball samples. From day 0–14, all fish balls exhibited no significant changes. However, during the end of the storage period, thyme and rosemary incorporated samples exhibited significant differences (*p* < 0.05) in comparison with the control and basil treatment for appearance, color, and general acceptance. In the case of basil samples, a lower score for odor and acceptance score is significantly different (*p* < 0.05) from the rest groups. The preservative properties of rosemary and thyme are known to retain sensory attributes, especially odor attributes, during storage [17]. The action is related to total phenolic composition, which is known to impart antioxidative ability, reducing lipid oxidation usually associated with off-odor evolution [27]. Especially thyme could mask the fish odor improving the sensory quality [28]. However, the combination of plant extracts and vacuum packing further enhanced the sensory quality from < 14 days to 28 days at chilled storage conditions, as reported by [19] for mackerel fish balls. This could be possibly attributed to the synergistic impact of plant extract with vacuum packaging.

Similarly, sensory scores for cooked fish balls showed lower sensory scores (color, odor, taste, texture, and general acceptance). However, the samples treated with thyme and rosemary were acceptable during the 28-day storage period. The lowered rate of lipid oxidation and microorganisms could possibly lessen the generation of volatile components during storage. Besides, the impact of cooking could further reduce the formation of undesirable compounds ^28^. The lowest sensory quality for samples treated with basil extracts could be attributed to lower total phenolic content, which could lower the antimicrobial and antioxidant activity of basil extract [14]. Additionally, the lower concentration of basil (0.5%) evaluated in this study could be the possible reason [14].

### 3.2. Chemical Analysis

Different results have been reported in various studies for fresh mackerel in terms of lipid oxidation parameters [29]. This could be attributed to the impacts of the catching period and feeding habit, causing variation in total lipid content [30] and impacts of processing conditions. Mackerel is regarded as a fatty fish susceptible to lipid oxidation [31].

#### 3.2.1. Peroxide Value (PV)

The PV value of mackerel balls with natural extracts packed under vacuum stored conditions for 28 days is given in Table 3. No significant difference was observed for PV among groups for 7 days (3.68–4.20 meq O_2_/kg), however, with further progress in storage, significant changes were observed. Further, no significant difference (*p* < 0.05) was observed between the control (5.60 meq O_2_/kg) and basil (5.34 meq O_2_/kg) treated sample. The results indicated the lower potential of basil extract to inhibit hydroperoxide formation in fishery products [14,16]. Significantly lower PV(*p* < 0.05) values were observed in thyme (4.35 meq O_2_/kg) and rosemary (4.38 meq O_2_/kg) treated fish balls. This might be attributed to the combined effects of the natural extracts (higher total phenolic content) and elimination of oxygen in vacuum packaging conditions on inhibiting the primary lipid oxidation during the 28-day storage. Similar findings were reported by the inclusion of natural extracts that is rich in phenolic content (free radical quencher and stabilize free radicals), vacuum packaging (eliminating oxygen), and combination [15,32,33]. However, all samples exhibited PV within the maximum permissible limits (30 meq O_2_/kg). The initial increase and decrease in PV values during storage could be due to the conversion of primary oxidation products to secondary oxidation compounds and correspond with the results of the TBA measurement [34].

#### 3.2.2. Thiobarbituricacid (TBA)

TBA values indicate the formation of secondary lipid oxidation products evaluated as the MDA content, indicating lipid oxidation in aquatic food products [35]. Mackerel is fatty fish and prone to oxidation, thus, TBA values for the control samples increased (2.18 to 2.78 mg MA/kg), which was not different (*p* < 0.05) from the basil treated sample (Table 3). Lipid oxidation propagated until day 11 and decreased on day 14; the decrease was highest in natural extract treated samples attributed to the ability of natural extract containing phenols to inhibit lipid oxidation and vacuum packaging, eliminating oxygen [32,33]. High phenolic composition is attributed to higher radical scavenging and reduced lipid peroxidation [6,10]. All samples retained malondialdehyde below the unacceptable limit of 5 mg/kg, which correlates with the action of natural extracts and packaging in vacuumed conditions [18]. The slight abrupt changes in TBA for samples during the initial storage period confirm the conclusion suggested by [36] regarding MDA formation from microbial growth. Rosemary-treated samples exhibited the lowest (2.24 mg MA/kg) TBA values (*p* < 0.05) during storage time. The ability of plant extract is known to inhibit lipid oxidation by diverse mechanisms (lower generation of free radical and quench free radical as well), lowering the formation of TBA [12]. The antioxidant characteristics of rosemary, thyme, and basil have been well indicated in different muscle food systems due to the presence of phenolic constituents exhibiting bioactivity [14,28]. The findings were in agreement with findings reported by [37] for fish balls made from hairtail fish using grape seed, sage, and oregano extracts. Additionally, vacuum packing exhibited synergism with natural extracts by lowering lipid oxidation, as reported in mackerel and rainbow trout [38].

#### 3.2.3. Free Fatty Acid (FFA)

The FFA profile for mackerel fish balls with natural extracts packed under vacuum during storage showed an increasing trend (Table 3). FFA are liberated by the hydrolysis of lipids by enzymatic action in the presence of water or oxidation [33] and are responsible for the alteration of sensory quality attributes in fish products [2]. FFA values increased in all samples; however, at the end of the storage period, no significant difference (*p* < 0.05) was observed amongst the samples. In contrast, the rise during the storage period was significant (*p* < 0.05) for all the samples. The increase could be attributed to enzymatic lipolysis of lipids and not oxidative damage due to vacuum packaging conditions but, the values were relatively smaller (<5.60% oleic acid). Results with slight variation were found by [28] for rainbow trout, clearly demonstrating the ability of vacuum packaging and lower temperature storage to reduce the deterioration regardless of natural extracts [32].

#### 3.2.4. Total Volatile Base Nitrogen (TVB-N)

The degradation of fish proteins by the action of microorganisms and enzymes generate volatile bases (ammonia, trimethyl amine, and dimethyl amine) known as TVB-N [2,3]. The evolution of TVB-N in mackerel fish balls made with different natural extracts during the storage period are presented in Table 4. No significant difference (*p* < 0.05) was observed in the evolution of TVB-N compounds during the initial period up to 11 days (20.66–16.52 mg N/100 g). Further increases in the storage period were significant (*p* < 0.05) amongst groups and the storage period. The TVB-N content was within the maximum permissible limits (35–40 mg N/100 g) for consumable safety. The maximum values were 23.28 mg N/100 g for the control and 21.64 mg N/100 g for basil treated sample; thus, the results were in agreement with data of sensorial rejection of raw fish balls. A significant reduction (*p* < 0.05) in TVB-N content for mackerel balls with rosemary (18.08 mg N/100 g) and thyme (18.08 mg N/100 g) content was found. The results indicated that the inclusion of natural extract having higher phenolic content combined with vacuum packaging significantly reduced the evolution of volatile amines (TVB-N) of fish balls compared to the control group [16]. The inhibition of TVB-N development is likely due to lowered deamination of non-proteinous nitrogenous compounds owing to decreased microbial growth [39]. The microbial inhibition could possibly be due to the antimicrobial effect of natural extracts and vacuum packaging in combination [15].

#### 3.2.5. pH Value

There was a significant difference (*p* < 0.05) in the changes inpH in all mackerel fish balls samples for different extracts during the storage period (Table 4). The increase in pH (6.19–6.57) could be due to the accumulation of alkaline compounds generated during the storage period [18]. The slight variation could be attributed to several processing and preservation impacts as well as vacuum packaging [19]. Plant extracts are also known to stabilize the pH and maintain it below 7 [37]. The increase inpH is attributed to the generation of volatile compounds owing to the degradation of muscle (autolysis or microorganisms) and is used as a spoilage indicator for fish products. All the samples were under the maximum permissible limits (7.6) suggested by [40]. The results were in line with findings (6.5–6.79) for battered and breaded products for catla fish as reported by Pawar and others [2]. Similarly, the effects of natural extracts maintained pH (6.89–7.21) for fish fillets during 24 days of storage [28].

### 3.3. Microbiological Quality

**Figure 1:**
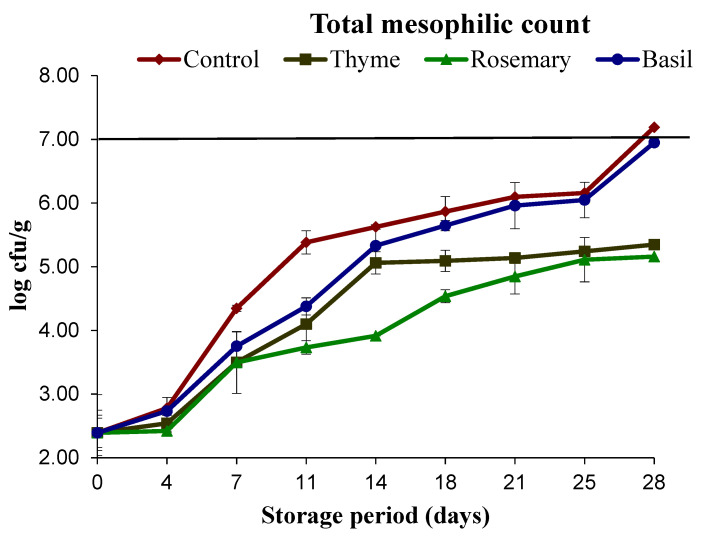
Changes in total mesophilic count from mackerel lballs during chilled storage. The horizontal line at 7 indicates maximum permissible limits.

Figure 1 shows the variation in the total mesophilic count for mackerel balls treated with natural extracts and under vacuum packaging. The lower initial count of mesophiles (2.39 log cfu/g) suggeststhe high microbial quality of the initial sample (ICMSF, 2011). However, the total mesophilic count for mackerel fish increased (*p* < 0.05) during storage (Figure 1). The control samples without any natural preservatives exceeded the maximum permissible limit (7 log cfu/g) (ICMSF, 2011) at the end of the storage period(28 days). Although microbial growth increased in all samples during storage, fish balls treated with natural extracts inhibited (*p* < 0.05) the increase in total mesophilic count, attributed to their remarkable antimicrobial activity and vacuum packaging [15]. The use of basil extract combined with vacuum packaging had similar results with control samples exhibiting only the impact of vacuum packaging. The vacuum environment is known to inhibit the growth of microorganisms due to the elimination of oxygen required for the survival of microbes [32]. Similarly, the impact of natural extracts (mint extract) in combination with vacuum packing inhibited the total viable count over air pack and vacuum pack samples [29]. The antimicrobial impact of natural plant extracts is due to the ability of phenolic compounds present in natural extracts to disturb the cell membrane and induce oxidative stress causing the lysis of microorganisms [6,10]. Additionally, vacuum packaging is known to prevent the growth of microorganisms due to lack of oxygen in packed samples and storage of samples at lower temperatures reduces the growth of anaerobic microorganisms as well.

## 4. Conclusions

Incorporation of thyme and rosemary extracts (0.05%) in mackerel balls when packed under vacuum retained their chemical (PV: <4.35 and 4.38; TBA: <2.34 and 2.24; FFA: <5.4 and 5.41), microbiological (inhibition in order of rosemary, thyme, and basil) and sensorial quality for 28 days when stored under chilled conditions (4 ± 2 °C). Basil extract and vacuum packaging had no impact on the quality of fish balls. Natural extracts were rich in phenolic content and exerted antimicrobial and antioxidative activity, which was further supported by vacuum packaging and lower temperature storage conditions. Amongst the evaluated extracts, thyme and rosemary could be recommended for preserving fish balls. Overall the results suggest the excellent ability of natural extracts to inhibit oxidation of lipids, microbial degradation, and improved sensory quality of mackerel fish balls, assuring seafood safety to consumers and shelf life extension to the processor.

## Figures and Tables

**Table 1:** Sensory quality changes of raw fishball during storage (4 ± 2 °C).

Sensory Parameters	Storage Time (Day)	Groups			
		Control % 0.05	Thyme % 0.05	Rosemary % 0.05	Basil % 0.05
Appearance	0	5.0 ± 0.00 ^aX^	5.0 ± 0.00 ^aX^	5.0 ± 0.00 ^aX^	5.0 ± 0.00 ^aX^
	4	5.0 ± 0.00 ^aX^	5.0 ± 0.00 ^aX^	5.0 ± 0.00 ^aX^	5.0 ± 0.00 ^aX^
	7	5.0 ± 0.00 ^aX^	5.0 ± 0.00 ^aX^	5.0 ± 0.00 ^aX^	5.0 ± 0.00 ^aX^
	11	4.1 ± 0.25 ^bX^	4.3 ± 0.29 ^bX^	4.4 ± 0.25 ^bX^	4.1 ± 0.25 ^bX^
	14	4.0 ± 0.00 ^bcX^	4.1 ± 0.25 ^bX^	4.1 ± 0.25 ^bcX^	4.0 ± 0.00 ^bX^
	18	3.6 ± 0.25 ^cY^	4.0 ± 0.00 ^bX^	4.0 ± 0.00 ^cX^	3.9 ± 0.25 ^bY^
	21	3.3 ± 0.00 ^dY^	3.5 ± 0.00 ^cX^	3.5 ± 0.00 ^dX^	3.5 ± 0.00 ^cX^
	25	3.0 ± 0.00 ^deY^	3.4 ± 0.25 ^cX^	3.4 ± 0.25 ^dX^	3.0 ± 0.00 ^dY^
	28	2.8 ± 0.29 ^eY^	3.2 ± 029 ^cX^	3.1 ± 0.25 ^dX^	2.6 ± 0.48 ^eY^
Color	0	5.0 ± 0.00 ^aX^	5.0 ± 0.00 ^aX^	5.0 ± 0.00 ^aX^	5.0 ± 0.00 ^aX^
	4	5.0 ± 0.00 ^aX^	5.0 ± 0.00 ^aX^	5.0 ± 0.00 ^aX^	5.0 ± 0.00 ^aX^
	7	4.5 ± 0.58 ^bX^	5.0 ± 0.00 ^aX^	5.0 ± 0.00 ^aX^	5.0 ± 0.00 ^aX^
	11	4.1 ± 0.25 ^bcX^	4.1 ± 0.25 ^bX^	4.3 ± 0.29 ^bX^	4.0 ± 0.00 ^bX^
	14	4.0 ± 0.00 ^cX^	4.0 ± 0.00 ^bX^	4.0 ± 0.00 ^bcX^	3.9 ± 0.25 ^bX^
	18	3.4 ± 0.25 ^dY^	3.9 ± 0.25 ^bcX^	3.9 ± 0.25 ^cdX^	3.8 ± 0.29 ^bXY^
	21	3.1 ± 0.25 ^deZ^	3.6 ± 0.25 ^cdX^	3.6 ± 0.25 ^deX^	3.4 ± 0.25 ^cXY^
	25	3.0 ± 0.00 ^efY^	3.4 ± 0.25 ^deX^	3.5 ± 0.00 ^efX^	3.0 ± 0.00 ^dY^
	28	2.6 ± 0.25 ^fY^	3.2 ± 0.29 ^eX^	3.3 ± 0.29 ^fX^	2.5 ± 0.41 ^eY^
Odor	0	5.0 ± 0.00 ^aX^	5.0 ± 0.00 ^aX^	5.0 ± 0.00 ^aX^	5.0 ± 0.00 ^aX^
	4	5.0 ± 0.00 ^aX^	5.0 ± 0.00 ^aX^	5.0 ± 0.00 ^aX^	4.5 ± 0.58 ^bX^
	7	4.5 ± 0.58 ^bX^	5.0 ± 0.00 ^aX^	5.0 ± 0.00 ^aX^	4.6 ± 0.48 ^aX^
	11	4.1 ± 0.25 ^bcY^	5.0 ± 0.00 ^aX^	5.0 ± 0.00 ^aX^	3.4 ± 0.25 ^cZ^
	14	4.0 ± 0.00 ^cY^	4.8 ± 0.29 ^aX^	4.8 ± 0.29 ^aX^	3.3 ± 0.29 ^cdZ^
	18	3.3 ± 0.29 ^dY^	4.0 ± 0.00 ^bX^	4.0 ± 0.00 ^bX^	3.1 ± 0.25 ^cdeY^
	21	3.1 ± 0.00 ^deY^	3.8 ± 0.29 ^cX^	3.9 ± 0.25 ^bcX^	3.1 ± 0.00 ^cdeY^
	25	3.0 ± 0.00 ^deY^	3.5 ± 0.00 ^dX^	3.6 ± 0.25 ^cX^	3.0 ± 0.00 ^deY^
	28	2.8 ± 0.29 ^eYZ^	3.3 ± 0.29 ^eXY^	3.1 ± 0.25 ^dX^	2.6 ± 0.25 ^eZ^
General acceptance	0	5.0 ± 0.00 ^aX^	5.0 ± 0.00 ^aX^	5.0 ± 0.00 ^aX^	5.0 ± 0.00 ^aX^
	4	5.0 ± 0.00 ^aX^	5.0 ± 0.00 ^aX^	5.0 ± 0.00 ^aX^	5.0 ± 0.00 ^aX^
	7	5.0 ± 0.00 ^aX^	5.0 ± 0.00 ^aX^	5.0 ± 0.00 ^aX^	5.0 ± 0.00 ^aX^
	11	4.1 ± 0.25 ^bX^	4.3 ± 0.29 ^bX^	4.3 ± 0.29 ^bX^	4.1 ± 0.25 ^bX^
	14	4.0 ± 0.00 ^bX^	4.0 ± 0.00 ^bcX^	4.0 ± 0.00 ^bcX^	3.9 ± 0.25 ^bcX^
	18	3.4 ± 0.25 ^cY^	3.9 ± 0.25 ^cdX^	3.9 ± 0.25 ^cX^	3.8 ± 0.29 ^cXY^
	21	3.1 ± 0.25 ^dZ^	3.8 ± 0.29 ^cdXY^	3.9 ± 0.25 ^cX^	3.4 ± 0.25 ^dYZ^
	25	3.0 ± 0.00 ^dY^	3.5 ± 0.00 ^dX^	3.5 ± 0.00 ^dX^	3.0 ± 0.00 ^eY^
	28	2.6 ± 0.25 ^eY^	3.1 ± 0.25 ^eX^	3.2 ± 0.29 ^dX^	2.6 ± 0.29 ^fY^

Mean values (*n* = 3) ±SD in same row with different letters (capitalized) represent significant difference (*p* < 0.05). Mean values 9*n* = 3) ±SD in same column with different letters (small) represent significant difference (*p* < 0.05).

**Table 2:** Sensory quality changes of cooked fishball during chilled (4 ± 2 °C) storage.

Storage Time (Day)	Groups	Color	Odor	Taste	Texture	General Acceptance
0	Control	9.00 ± 0.00 ^ax^	9.00 ± 0.00 ^ax^	9.00 ± 0.00 ^ax^	9.00 ± 0.00 ^ax^	9.00 ± 0.00 ^ax^
	Tymus	9.00 ± 0.00 ^ax^	9.00 ± 0.00 ^ax^	9.00 ± 0.00 ^ax^	9.00 ± 0.00 ^ax^	9.00 ± 0.00 ^ax^
	Rosemary	9.00 ± 0.00 ^ax^	9.00 ± 0.00 ^ax^	9.00 ± 0.00 ^ax^	9.00 ± 0.00 ^ax^	9.00 ± 0.00 ^ax^
	Basil	9.00 ± 0.00 ^ax^	9.00 ± 0.00 ^ax^	9.00 ± 0.00 ^ax^	9.00 ± 0.00 ^ax^	9.00 ± 0.00 ^ax^
4	Control	8.33 ± 0.82 ^bX^	8.17 ± 0.75 ^bX^	8.33 ± 0.82 ^bX^	8.33 ± 0.82 ^bX^	8.33 ± 0.82 ^bX^
	Tymus	8.67 ± 0.52 ^bX^	8.50 ± 0.55 ^abX^	8.50 ± 0.84 ^abX^	8.50 ± 0.55 ^abX^	8.50 ± 0.55 ^abX^
	Rosemary	8.67 ± 0.52 ^abX^	8.67 ± 0.52 ^abX^	8.50 ± 0.55 ^abX^	8.67 ± 0.52 ^abX^	8.67 ± 0.52 ^abX^
	Basil	8.50 ± 0.55 ^abX^	8.50 ± 0.84 ^aX^	8.00 ± 0.89 ^bX^	8.67 ± 0.52 ^aX^	8.50 ± 0.55 ^aX^
7	Control	8.17 ± 0.41 ^bcX^	8.00 ± 0.63 ^bXY^	7.83 ± 0.41 ^bX^	7.67 ± 0.52 ^cX^	7.83 ± 0.41 ^bcXY^
	Tymus	8.17 ± 0.75 ^bcX^	8.33 ± 0.82 ^abXY^	8.17 ± 0.41 ^bcX^	8.33 ± 0.52 ^bcX^	8.17 ± 0.75 ^bXY^
	Rosemary	8.33 ± 0.52 ^bcX^	8.58 ± 0.49 ^abX^	8.33 ± 0.82 ^abX^	8.17 ± 0.75 ^bcX^	8.33 ± 0.52 ^bcX^
	Basil	8.17 ± 0.41 ^bcX^	7.50 ± 0.84 ^bcY^	6.67 ± 0.82 ^cY^	7.83 ± 0.41 ^bX^	7.50 ± 0.84 ^bY^
11	Control	8.00 ± 0.63 ^bcX^	7.83 ± 0.75 ^bX^	7.25 ± 0.42 ^cY^	7.50 ± 0.84 ^cdX^	7.33 ± 0.52 ^cdY^
	Tymus	8.00 ± 0.00 ^cdX^	8.17 ± 0.41 ^bX^	8.00 ± 0.00 ^bcdX^	8.00 ± 0.63 ^bcdX^	8.08 ± 0.20 ^bX^
	Rosemary	8.17 ± 0.41 ^bcX^	8.17 ± 0.41 ^bX^	8.08 ± 0.20 ^bcX^	8.08 ± 0.66 ^bcdX^	8.17 ± 0.41 ^bcX^
	Basil	7.83 ± 0.75 ^cdX^	7.67 ± 0.52 ^bX^	6.50 ± 0.55 ^cZ^	7.75 ± 0.42 ^bcX^	7.17 ± 0.75 ^bcY^
14	Control	7.67 ± 0.52 ^cdX^	7.67 ± 0.52 ^bX^	7.17 ± 0.41 ^cY^	7.33 ± 0.52 ^cdY^	7.17 ± 0.41 ^dY^
	Tymus	7.92 ± 0.20 ^cdX^	8.00 ± 0.00 ^bX^	7.83 ± 0.41 ^cdeX^	7.83 ± 0.41 ^cdXY^	8.00 ± 0.00 ^bcX^
	Rosemary	7.83 ± 0.41 ^cdX^	8.00 ± 0.63 ^bX^	7.83 ± 0.75 ^bcdX^	8.00 ± 0.00 ^cdX^	7.92 ± 0.20 ^cdX^
	Basil	7.67 ± 0.52 ^cdX^	6.83 ± 0.75 ^cdY^	6.17 ± 0.41 ^cdZ^	7.33 ± 0.52 ^cdY^	7.00 ± 0.63 ^bcY^
18	Control	7.33 ± 0.52 ^deX^	6.83 ± 0.41 ^cXY^	6.67 ± 0.52 ^cdY^	7.25 ± 0.42 ^cdX^	7.08 ± 0.20 ^dXY^
	Tymus	7.42 ± 0.49 ^deX^	7.17 ± 0.98 ^cX^	7.50 ± 0.55 ^deX^	7.50 ± 0.55 ^deX^	7.50 ± 0.55 ^cdX^
	Rosemary	7.50 ± 0.55 ^dX^	7.33 ± 0.52 ^cX^	7.50 ± 0.55 ^cdX^	7.50 ± 0.55 ^deX^	7.50 ± 0.55 ^deX^
	Basil	7.33 ± 0.52 ^deX^	6.33 ± 0.52 ^dY^	5.67 ± 0.41 ^deZ^	7.17 ± 0.26 ^deX^	6.92 ± 0.20 ^bcY^
21	Control	7.00 ± 0.00 ^eX^	6.67 ± 0.52 ^cXY^	6.33 ± 0.52 ^dY^	7.00 ± 0.00 ^dXY^	6.42 ± 0.49 ^eZ^
	Tymus	7.25 ± 0.42 ^eX^	7.08 ± 0.20 ^cX^	7.25 ± 0.42 ^eX^	7.25 ± 0.27 ^eX^	7.25 ± 0.27 ^dXY^
	Rosemary	7.33 ± 0.52 ^dX^	7.25 ± 0.88 ^cX^	7.33 ± 0.52 ^deX^	7.33 ± 0.52 ^eX^	7.33 ± 0.52 ^eX^
	Basil	7.00 ± 0.00 ^eX^	6.17 ± 0.41 ^dY^	5.33 ± 0.52 ^eZ^	6.83 ± 0.26 ^eY^	6.75 ± 0.42 ^cYZ^
25	Control	6.00 ± 0.00 ^fX^	5.67 ± 0.52 ^dY^	5.33 ± 0.52 ^eY^	6.00 ± 0.00 ^eZ^	5.33 ± 0.52 ^fY^
	Tymus	6.25 ± 0.42 ^fX^	6.58 ± 0.49 ^cdX^	6.25 ± 0.42 ^fX^	6.58 ± 0.49 ^fY^	6.67 ± 0.52 ^eX^
	Rosemary	6.33 ± 0.52 ^eX^	7.00 ± 0.00 ^cX^	6.67 ± 0.52 ^efX^	7.00 ± 0.00 ^eX^	7.00 ± 0.00 ^eX^
	Basil	6.17 ± 0.41 ^fX^	5.33 ± 0.52 ^eY^	5.25 ± 0.42 ^eY^	6.08 ± 0.20 ^fZ^	5.42 ± 0.49 ^dY^
28	Control	5.75 ± 0.42 ^fX^	4.67 ± 0.52 ^eY^	4.33 ± 0.52 ^fY^	5.83 ± 0.41 ^eXY^	4.83 ± 0.41 ^fY^
	Tymus	6.08 ± 0.80 ^fX^	6.17 ± 0.68 ^dX^	6.17 ± 0.75 ^fX^	6.33 ± 0.52 ^fX^	6.25 ± 0.61 ^eX^
	Rosemary	6.00 ± 0.89 ^eX^	6.17 ± 0.75 ^dX^	6.17 ± 0.75 ^fX^	6.17 ± 0.75 ^fXY^	6.17 ± 0.75 ^fX^
	Basil	5.67 ± 0.52 ^fX^	4.50 ± 0.55 ^fY^	4.50 ± 0.55 ^fY^	5.58 ± 0.49 ^gY^	4.92 ± 0.20 ^dY^

Mean values (*n* = 3) ±SD in same row with different letters (capitalized) represent significant difference (*p* < 0.05). Mean values 9*n* = 3) ±SD in same column with different letters (small) represent significant difference (*p* < 0.05).

**Table 3:** Changes in lipid oxidation TBA, FFA, and PV content of fishball during chilled (4 ± 2 °C) storage.

Storage Days	Control	Thymus	Rosemary	Basil
**PV (meq O_2_/kg)**				
0	4.34 ± 0.07 ^cdX^	4.34 ± 0.07 ^bX^	4.34 ± 0.07 ^bcX^	4.34 ± 0.07 ^bX^
4	3.51 ± 0.21 ^eX^	3.45 ± 0.01 ^cX^	3.23 ± 0.21 ^dX^	3.20 ± 0.44 ^dX^
7	4.20 ± 0.31 ^dX^	3.68 ± 0.88 ^bcX^	3.84 ± 0.19 ^cX^	3.79 ± 0.52 ^cX^
11	4.77 ± 0.19 ^bcX^	3.71 ± 0.32 ^bcY^	3.06 ± 0.14 ^dZ^	3.65 ± 0.31 ^cdY^
14	5.88 ± 0.29 ^aX^	3.85 ± 0.19 ^bcY^	3.87 ± 0.58 ^cY^	3.77 ± 0.02 ^cY^
18	5.65 ± 0.14 ^aX^	5.47 ± 0.38 ^cXY^	5.19 ± 0.17 ^aY^	5.06 ± 5.06 ^aY^
21	4.63 ± 0.04 ^bcdX^	4.02 ± 0.20 ^bcY^	4.77 ± 0.10 ^abX^	4.57 ± 0.09 ^bX^
25	4.92 ± 0.29 ^bX^	4.32 ± 0.30 ^bY^	4.44 ± 0.14 ^bXY^	4.28 ± 0.23 ^bY^
28	5.60 ± 0.44 ^aX^	4.35 ± 0.07 ^bZ^	4.38 ± 0.59 ^abYZ^	5.34 ± 0.21 ^aXY^
**TBA (mg MA/kg)**				
0	2.18 ± 0.08 ^deX^	2.18 ± 0.08 ^bcX^	2.18 ± 0.08 ^bcX^	2.18 ± 0.08 ^cX^
4	3.37 ± 0.22 ^aX^	2.58 ± 0.03 ^aY^	2.67 ± 0.05 ^aY^	2.61 ± 0.13 ^bY^
7	3.21 ± 0.41 ^aX^	2.43 ± 0.35 ^aY^	1.85 ± 0.04 ^dZ^	2.74 ± 0.11 ^abY^
11	2.44 ± 0.21 ^cdY^	2.02 ± 0.13 ^cZ^	1.87 ± 0.12 ^dT^	2.76 ± 0.04 ^abX^
14	2.04 ± 0.14 ^eX^	1.61 ± 0.10 ^dY^	1.52 ± 0.06 ^eY^	1.62 ± 0.03 ^dY^
18	2.46 ± 0.13 ^cdY^	2.44 ± 0.10 ^aY^	2.11 ± 0.17 ^cZ^	3.02 ± 0.14 ^aX^
21	2.51 ± 0.10 ^bcXY^	2.39 ± 0.09 ^abY^	2.36 ± 0.12 ^bY^	2.87 ± 0.55 ^abX^
25	2.70 ± 0.13 ^bcX^	2.46 ± 0.09 ^aXY^	2.31 ± 0.16 ^bY^	2.58 ± 0.25 ^bXY^
28	2.78 ± 0.15 ^bX^	2.43 ± 0.21 ^aXY^	2.24 ± 0.24 ^bcY^	2.63 ± 0.28 ^bX^
**FFA (% Oleic acid)**				
0	4.07 ± 0.09 ^fX^	4.07 ± 0.09 ^cX^	4.07 ± 0.09 ^dX^	4.07 ± 0.09 ^cX^
4	4.25 ± 0.15 ^efX^	4.11 ± 0.03 ^cX^	4.17 ± 0.12 ^cdX^	4.20 ± 0.31 ^cX^
7	4.43 ± 0.13 ^deX^	4.26 ± 0.12 ^cX^	4.23 ± 0.15 ^cdX^	4.30 ± 0.29 ^cX^
11	4.52 ± 0.07 ^dX^	4.37 ± 0.44 ^bcX^	4.30 ± 0.09 ^cX^	4.43 ± 0.12 ^cX^
14	5.13 ± 0.18 ^cX^	4.93 ± 0.02 ^abX^	4.86 ± 0.20 ^bX^	4.92 ± 0.06 ^bX^
18	5.32 ± 0.23 ^bcX^	5.03 ± 0.12 ^aX^	5.24 ± 0.16 ^aX^	5.28 ± 0.18 ^abX^
21	5.36 ± 0.10 ^abcX^	5.28 ± 0.59 ^aX^	5.27 ± 0.09 ^aX^	5.12 ± 0.22 ^abX^
25	5.41 ± 0.04 ^abX^	5.34 ± 0.71 ^aX^	5.33 ± 0.09 ^aX^	5.36 ± 0.39 ^abX^
28	5.60 ± 0.17 ^aX^	5.40 ± 0.09 ^aX^	5.41 ± 0.19 ^aX^	5.49 ± 0.28 ^aX^

Mean values (*n* = 3) ±SD in same row with different letters (capitalized) represent significant difference (*p* < 0.05). Mean values 9*n* = 3) ±SD in same column with different letters (small) represent significant difference (*p* < 0.05).

**Table 4:** TBV-N and pH values of fishball during chilled (4 ± 2 °C) storage.

Storage Months	Control	Thyme	Rosemary	Basil
**TVB-N (mg/100 g)**				
0	16.52 ± 0.42 ^eX^	16.52 ± 0.42 ^eX^	16.52 ± 0.42 ^eX^	16.52 ± 0.42 ^dX^
4	21.16 ± 0.42 ^cX^	19.10 ± 1.05 ^cdY^	19.97 ± 1.13 ^aXY^	20.46 ± 0.81 ^bcXY^
7	19.11 ± 1.74 ^dXY^	19.26 ± 0.38 ^cdXY^	18.17 ± 0.06 b^cdY^	20.25 ± 0.70 ^cX^
11	20.46 ± 0.33 ^cdX^	19.43 ± 1.37 ^cdX^	20.01 ± 0.78 ^aX^	20.66 ± 0.41 ^bcX^
14	20.25 ± 0.48 ^cdXY^	20.77 ± 1.12 ^bcX^	19.37 ± 0.32 ^abY^	20.67 ± 0.17 ^bcX^
18	25.57 ± 0.86 ^aX^	22.77 ± 1.56 ^aY^	16.98 ± 0.38 ^deZ^	25.15 ± 0.04 ^aX^
21	25.28 ± 1.48 ^aX^	21.63 ± 1.17 ^abY^	17.66 ± 0.77 ^cdeZ^	25.34 ± 0.98 ^aX^
25	19.96 ± 0.33 ^cdX^	18.08 ± 0.04 ^deY^	18.33 ± 1.07 ^bcY^	20.01 ± 0.83 ^cX^
28	23.28 ± 1.06 ^bX^	18.92 ± 0.15 ^dZ^	18.08 ± 0.05 ^cdZ^	21.64 ± 0.78 ^bY^
**pH**				
0	6.22 ± 0.08 ^eA^	6.22 ± 0.08 ^dA^	6.22 ± 0.08 ^dA^	6.22 ± 0.08 ^fA^
4	6.39 ± 0.02 ^bcC^	6.47 ± 0.01 ^bA^	6.45 ± 0.01 ^bAB^	6.44 ± 0.01 ^abB^
7	6.19 ± 0.01 ^eC^	6.53 ± 0.02 ^aA^	6.44 ± 0.09 ^bB^	6.47 ± 0.02 ^aB^
11	6.31 ± 0.05 ^cdC^	6.40 ± 0.03 ^cB^	6.46 ± 0.01 ^bA^	6.44 ± 0.01 ^abAB^
14	6.29 ± 0.03 ^dB^	6.38 ± 0.01 ^cA^	6.41 ± 0.01 ^bA^	6.38 ± 0.02 ^cdA^
18	6.44 ± 0.03 ^abA^	6.42 ± 0.01 ^bcA^	6.42 ± 0.01 ^bA^	6.42 ± 0.02 ^abcA^
21	6.48 ± 0.06 ^aA^	6.40 ± 0.01 ^cB^	6.30 ± 0.06 ^cC^	6.36 ± 0.02 ^deBC^
25	6.30 ± 0.09 ^cdB^	6.42 ± 0.02 ^cA^	6.43 ± 0.01 ^bA^	6.40 ± 0.01 ^bcdAB^
28	6.42 ± 0.00 ^abB^	6.57 ± 0.04 ^eD^	6.54 ± 0.02 ^aA^	6.32 ± 0.05 ^dC^

Mean values (*n* = 3) ±SD for TVB-N and (*n* = 4) ±SD for pH, in same row with different letters (capitalized) represent significant difference (*p* < 0.05). Mean values 9*n* = 3) ±SD in same column with different letters (small) represent significant difference (*p* < 0.05).

## Data Availability

Data is contained within the article.
